# Minimizing Computation and Communication Costs of Two-Sided Secure Distributed Matrix Multiplication under Arbitrary Collusion Pattern

**DOI:** 10.3390/e26050407

**Published:** 2024-05-08

**Authors:** Jin Li, Nan Liu, Wei Kang

**Affiliations:** 1 National Mobile Communications Research Laboratory, Southeast University, Nanjing 211189, China; lijin@seu.edu.cn (J.L.); nanliu@seu.edu.cn (N.L.); 2 School of Information Science and Engineering, Southeast University, Nanjing 211189, China

**Keywords:** secure distributed matrix multiplication, arbitrary collusion pattern, integer linear programming, integer geometric programming

## Abstract

This paper studies the problem of minimizing the total cost, including computation cost and communication cost, in the system of two-sided secure distributed matrix multiplication (SDMM) under an arbitrary collusion pattern. In order to perform SDMM, the two input matrices are split into some blocks, blocks of random matrices are appended to protect the security of the two input matrices, and encoded copies of the blocks are distributed to all computing nodes for matrix multiplication calculation. Our aim is to minimize the total cost, overall matrix splitting factors, number of appended random matrices, and distribution vector, while satisfying the security constraint of the two input matrices, the decodability constraint of the desired result of the multiplication, the storage capacity of the computing nodes, and the delay constraint. First, a strategy of appending zeros to the input matrices is proposed to overcome the divisibility problem of matrix splitting. Next, the optimization problem is divided into two subproblems with the aid of alternating optimization (AO), where a feasible solution can be obtained. In addition, some necessary conditions for the problem to be feasible are provided. Simulation results demonstrate the superiority of our proposed scheme compared to the scheme without appending zeros and the scheme with no alternating optimization.

## 1. Introduction

With the development of the Internet of Things (IoT), the ubiquitous wireless devices can generate massive data via environment monitoring or target tracking [1]. However, due to the limited power or hardware architecture, these wireless devices cannot satisfy the data processing and computation requirements by themselves. This inspires wireless devices to seek help from online computing nodes who can assist in computation and data processing. Furthermore, distributed computing nodes can be employed to further accelerate the computation and data processing tasks, which means wireless devices can assign computation tasks to many different computing nodes, e.g., Apache Spark [2] and MapReduce [3]. On the other hand, if the online computing nodes are untrustworthy, we should also guarantee data security. Hence, how to perform computation of data with the aid of distributed computing nodes in a secure fashion is an important problem.

In this paper, we focus on the secure distributed matrix multiplication (SDMM) problem [4,5,6,7,8]. In [7,8], the trace-mapping framework has been employed to achieve communication-efficient schemes in the SDMM. The authors of [9] proposed a model of SDMM from an information-theoretic perspective. The user wishes to compute the product of two input matrices A and B with the aid of distributed computing nodes while guaranteeing the security of the information about the two input matrices. Two cases are considered: one-sided security and two-sided security. In the first case, the user only wants to protect the information security of matrix A, and B is a public matrix known to all computing nodes [10]. In the second case, we need to consider the information security of both matrices A and B [9,11]. The information theft by the distributed computing nodes can be modeled by the collusion pattern, which has also been studied in problems of secret sharing [12] and private information retrieval [13,14]. Some of the existing literature has studied the SDMM problem under homogeneous collusion patterns, where up to *l* computing nodes may collude to obtain the information of the two input matrices [9,15,16,17,18]. To balance the tradeoff between the uplink and downlink cost, the works proposed two schemes based on the secure cross subspace alignment [15]. In [9], the authors characterized the fundamental limits of minimum communication overhead for the SDMM problem under homogeneous collusion pattern. The work in [16] proposed a scheme based on the polynomial codes on sub-tasks assigned to computing nodes, which can mitigate the straggling effects efficiently. In [18], the authors have adopted some random matrices to encode two input matrices for the purpose of meeting the requirement of security. Then, many encoded copies are sent to different computing nodes for computation. Finally, the user receives these computation results from computing nodes and recovers the product of the two input matrices. It has considered two cases: (1) encoding the input matrices without extra random matrices, i.e., generalized polydot code, and (2) encoding the input matrices with some random matrices to satisfy the security constraint, i.e., secure the generalized polydot code. They also show the superiority of the proposed scheme on the recovery threshold, i.e., the number of computation results that is needed for users to decode the desired result without error, and the communication load between the user and computing nodes, i.e., the amount of downloaded information from computing nodes. Recently, rather than focusing on the homogeneous collusion pattern, ref. [19] studied the SDMM problem under the arbitrary collusion pattern. Considering the two proposed performance metrics, i.e., the normalized download cost and normalized upload cost, they provide the optimal scheme for the one-sided SDMM problem and an achievable scheme for the two-sided SDMM problem.

Both the private information retrieval and SDMM problem considered in [14,19] deal with the non-homogeneous collusion pattern scenario. The common approach of these two problems is assigning different number of copies to different servers. Intuitively speaking, the servers that collude more will be assigned a lower number of copies. More specifically, in [14], the authors considered the ratio between the message size and the amount of downloaded information from the servers. Then, the work of [19] studied the SDMM problem under the arbitrary collusion pattern for a fixed matrix splitting factor, and different numbers of copies were distributed to different computing nodes based on the collusion pattern to minimize the performance of normalized download and upload costs. However, the heterogeneity of the computing nodes in terms of storage capacity, communication capability, and computing capability was not taken into consideration. When full heterogeneity is taken into consideration, the numbers of copies assigned to different servers will not only depend on its colluding behavior but also on its storage capacity, communication capability, and computing capability. Furthermore, the fixed matrix splitting factor may affect the performance of SDMM. Hence, in this work, we study the problem of two-sided SDMM under an arbitrary collusion pattern with the flexible matrix splitting factor. Furthermore, in order to measure the communication and computation performance of the system, a new performance metric called the total cost, which is composed of the computation cost and communication cost, has been proposed in our paper. Additionally, the storage capability of the computing nodes and the delay requirement of the user are also considered. Then, an optimization problem is formulated by minimizing the total cost, subject to the security constraint of the two input matrices, the decodability constraint of the desired result of the multiplication, the storage capacity of the computing nodes, and the delay constraint. In order to overcome the divisibility problem of matrix splitting, we also propose a strategy of appending zeros to the input matrices and discuss the feasible set of some matrix splitting factors for the optimality of the problem. Finally, an alternating optimization (AO) algorithm based on some solvers is adopted to obtain a feasible solution, and some necessary conditions for the feasibility of problem have been provided.

The contributions of our paper are summarized as follows:

- We propose a new performance metric, the total cost, which includes communication cost and computation cost, to measure the performance of the SDMM problem under arbitrary collusion pattern. Our aim is to minimize the total cost, overall matrix splitting factors, number of appended random matrices, and distribution vector, while satisfying the security constraint of the two input matrices, the decodability constraint of the desired result of the multiplication, the storage capacity of the computing nodes, and the delay constraint.
- To overcome the divisibility problem of matrix splitting, we propose a strategy of padding zeros to the input matrices, which can split the input matrices into an arbitrary number of blocks compared to the scheme without appending zeros. Moreover, the value ranges of some matrix splitting factors are discussed for the optimality of the problem.
- The formulated optimization problem is solved by an AO algorithm based on some solvers. More specifically, for the optimization subproblem corresponding to number of appended random matrices and distribution vector, the relationship between number of appended random matrices and distribution vector can be found so that the subproblem is transformed into an integer linear programming over the distribution vector, which can be solved by the MATLAB function “intlinprog”. Furthermore, we also provide some necessary conditions to verify the feasibility of this subproblem. Then, for the optimization subproblem corresponding to all matrix splitting factors, by relaxing the ceiling function and integer constraints, the subproblem can be transformed into an integer geometric programming problem solved by using “YALMIP”. Simulation results show that our proposed scheme with padding zeros is superior to the scheme without appending zeros and the scheme with no alternating optimization.

The rest of this paper is organized as follows: Section 2 introduces the system model of the two-sided SDMM under arbitrary collusion pattern. Section 3 proposes a zero-padding strategy, discusses the feasible set of some matrix splitting factors, and formulates an optimization problem. Section 4 provides the algorithm to solve the problem. Simulation results and conclusions are shown in Section 5 and Section 6, respectively.

**Notation 1.**  *In this paper, the following notations are used. $\left[1:N\right]$ denotes the set {1,2,⋯,N}. $h_{n}$ represents the n-th column vector of the matrix h. $1_{N}$ denotes the N×1 column vector. Positive integer is represented by $Z^{+}$, natural number is denoted by N, and the ceiling function is denoted by $\left\lceil \cdot \right\rceil$.*

## 2. System Model

**Figure 1:**
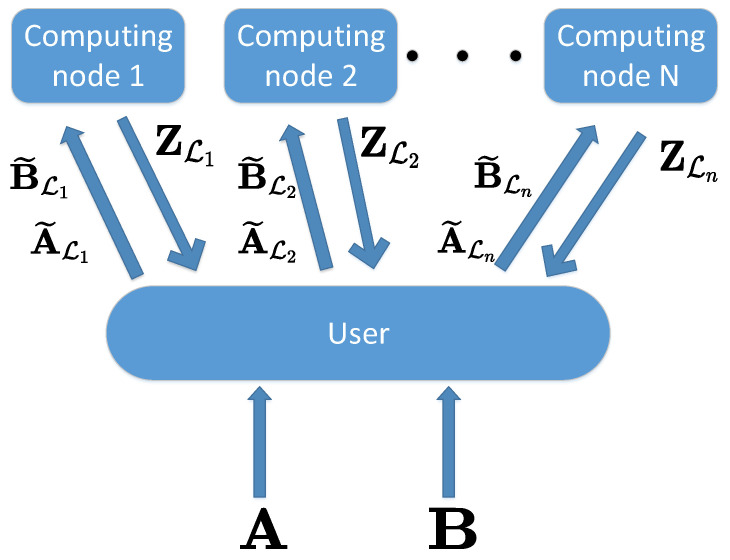
Two-sided secure distributed matrix multiplication.

As shown in Figure 1, we consider a user who wants to calculate the multiplication of two input matrices $A\in F^{T\times S}$ and $B\in F^{S\times D}$. We suppose that T,S and *D* are all integers and the finite field F is sufficiently large. Due to its own limited computational ability, the user wishes to split the two matrices A and B into many blocks and upload them to *N* computing nodes for computation. At the same time, both matrices A and B contain sensitive information, and the user does not want to leak any information to the *N* computing nodes.

We study the case where the computing nodes may collude with others to obtain information about the two matrices A and B. We represent the colluding behaviors by a collusion pattern P, which contains *M* colluding sets, i.e., $P=\{T_{1},T_{2},\cdots T_{M}\}$. Here, $T_{m}\subseteq \left[1:N\right]$ is the *m*-th colluding set, which means that computing nodes in $T_{m}$ may collude to obtain the information of the two matrices. We make the following two assumptions about the collusion pattern P:

(1) For ease of presentation, we only include the maximal colluding set in P. For instance, a colluding set {3,4,5,6} means that computing nodes 3, 4, 5, and 6 collude. This implies that computing nodes belonging to any subset of {3,4,5,6} also collude. However, for ease of presentation, we do not include the subsets of {3,4,5,6} in P.
(2) Every computing node must appear in at least one colluding set. This is because we assume that all computing nodes are curious, and no computing node can be trusted with the sensitive information of A and B.

A collusion pattern P can be represented by its incidence matrix $B_{P}$, of size N×M, i.e., if computing node *i* in the *j*-th colluding set of P, the value of the (i,j)-th element in $B_{P}$ is 1. For example, when P={{1,2,3},{1,4},{2,4},{3,4},{5}}, its incidence matrix is

(1) $$\begin{array}{c} B_{P}=\left[\begin{array}{ccccc} 1 & 1 & 0 & 0 & 0\\ 1 & 0 & 1 & 0 & 0\\ 1 & 0 & 0 & 1 & 0\\ 0 & 1 & 1 & 1 & 0\\ 0 & 0 & 0 & 0 & 1 \end{array}\right]. \end{array}$$

Due to the need to keep the two matrices secure, the user must encode A,B before uploading them to the computing nodes for computation. Assume that there are $N_{1}$ encoded copies with $N_{1}\geq N$, then these encoding functions are denoted as: $f=\left(f_{1},f_{2},\cdots ,f_{N_{1}}\right),g=\left(g_{1},g_{2},\cdots ,g_{N_{1}}\right).$ We use ${\tilde{A}}_{i}$ and ${\tilde{B}}_{i}$ to represent the *i*-th encoded copy of matrices A and B, respectively, $i\in [1:N_{1}]$, i.e., ${\tilde{A}}_{i}=f_{i}\left(A\right),{\tilde{B}}_{i}=g_{i}\left(B\right).$ The user distributes a subset of the encoded matrices to computing node *n*, where the indices of this subset are written as $L_{n}$, $L_{n}\subseteq \left[1:N_{1}\right]$. This is termed *the upload phase.*

The computing node *n* computes the product, i.e., $Z_{i}={\tilde{A}}_{i}{\tilde{B}}_{i}$, $i\in L_{n}$. Then, computing node *n* would send the computed results $Z_{i}$, $i\in L_{n}$ back to the user. This is termed *the download phase*.

In order to ensure the security of matrices A and B, the following security constraint must be satisfied,

(2) $$\begin{array}{c} I\left(A,B;{\left({\tilde{A}}_{L_{n}},{\tilde{B}}_{L_{n}}\right)}_{n\in T_{m}}\right)=0,\;\forall m\in \left[1:M\right]. \end{array}$$

which indicates that computing nodes in each colluding set, when putting their received copies together, can not obtain any information about the two matrices.

In addition, the user must be able to decode the desired product C=AB from the answers received from all the computing nodes, i.e., the decodability constraint

(3) $$\begin{array}{c} H(\mathrm{AB}|Z_{1},Z_{2},\cdots ,Z_{N_{1}})=0 \end{array}$$

must be satisfied.

### 2.1. Matrix Encoding Scheme

We use the secure generalized polydot code (SGPD) in [18] to encode the two input matrices. First, we split A into t×s blocks, while B can be split into s×d blocks, i.e.,

(4) $$A=\left[\begin{array}{ccc} A_{1,1} & \cdots \; & A_{1,s}\\ \vdots & \ddots & \vdots \\ A_{t,1} & \cdots & A_{t,s} \end{array}\right],\;B=\left[\begin{array}{ccc} B_{1,1} & \cdots \; & B_{1,d}\\ \vdots & \ddots & \vdots \\ B_{s,1} & \cdots & B_{s,d} \end{array}\right],$$

where *T* is divisible by *t*, *S* is divisible by *s*, and *D* is divisible by *d*. Then, $A_{i,j}$ is of size $t_{0}\times s_{0}$, and $B_{i,j}$ is of size $s_{0}\times d_{0}$, where we have defined

(5) $$\begin{array}{c} t_{0}=\frac{T}{t},\;s_{0}=\frac{S}{s},\;d_{0}=\frac{D}{d}. \end{array}$$

In view of the security constraint (2), we append some random matrices $K_{i,j}\in F^{\left(T/t)\times (S/s\right)}$ and $K_{i,j}^{\prime }\in F^{\left(S/s)\times (D/d\right)}$ as

(6) $$\begin{array}{cc} A^{*} & =\left[\begin{array}{ccc} A_{l,1} & \cdots \; & A_{1,s}\\ \vdots & \ddots & \vdots \\ A_{t,1} & \cdots & A_{t,s}\\ K_{1,1} & \cdots \; & K_{1,s}\\ \vdots & \ddots & \vdots \\ K_{l_{\Delta },1} & \cdots & K_{l_{\Delta },s} \end{array}\right], \end{array}$$

(7) $$\begin{array}{cc} B^{*} & =\left[\begin{array}{cccccc} B_{1,1} & \cdots \; & B_{1,d} & K_{1,1}^{\prime } & \cdots \; & K_{1,l_{\Delta }}^{\prime }\\ \vdots & \ddots & \vdots & \vdots & \ddots & \vdots \\ B_{s,1} & \cdots & B_{s,d} & K_{s,1}^{\prime } & \cdots \; & K_{s,l_{\Delta }}^{\prime } \end{array}\right], \end{array}$$

where $l_{\Delta }$ rows of random matrices are appended to matrix A, and $l_{\Delta }$ columns of random matrices are appended to matrix B, where $l_{\Delta }$ is a positive integer. Each element of the random matrices $K_{i,j}$ and $K_{i,j}^{\prime }$ are generated in an i.i.d. fashion according to the uniform distribution on F. Note that (6) and (7) are just one way of appending random matrices. The other case is given by Method 2 in [19]. For simplicity, we only study the case of (6) and (7), and the other case of appending random matrices can be treated in a similar fashion.

In this case, the encoded matrices are generated according to

(8) $$\begin{array}{cc} {\tilde{A}}_{i} & =\sum_{j=1}^{t}\sum_{k=1}^{s}A_{j,k}^{*}x_{i}^{s(j-1)+k-1}\\ & +\sum_{j=t+1}^{t^{*}}\sum_{k=1}^{s}A_{j,k}^{*}x_{i}^{s(j-1)+k-1}\;i=1,\cdots ,N_{1}, \end{array}$$

(9) $$\begin{array}{cc} {\tilde{B}}_{i} & =\sum_{k=1}^{s}\sum_{p=1}^{d}B_{k,p}^{*}x_{i}^{s-k+t^{*}s\left(p-1\right)}\\ & +\sum_{k=1}^{s}\sum_{p=d+1}^{d^{*}}B_{k,p}^{*}x_{i}^{t^{*}sd+s\left(p-d\right)-k}\;i=1,\cdots ,N_{1}, \end{array}$$

where $x_{i}$, $i=1,2,\cdots ,N_{1}$ are $N_{1}$ distinct non-zero elements in F, and we have defined $t^{*}=t+l_{\Delta },d^{*}=d+l_{\Delta }$.

The $N_{1}$ generated encoded copies of (8) and (9), i.e., $\left({\tilde{A}}_{i},{\tilde{B}}_{i}\right)$, $i\in [1:N_{1}]$, will be distributed to the computing nodes, where computing node *n* will receive ${\tilde{A}}_{L_{n}}$ and ${\tilde{B}}_{L_{n}}$, where $L_{n}\subseteq \left[1:N_{1}\right]$ is the index set of the encoded matrices distributed to computing node *n*. We assume that $L_{n},n\in \left[1:N\right],$ form a partition of the set $\left[1:N_{1}\right]$, which means that each encoded copy will be distributed to one and only one computing node. Upon receiving ${\tilde{A}}_{L_{n}}$ and ${\tilde{B}}_{L_{n}}$, computing node *n* will calculate $Z_{i}={\tilde{A}}_{i}{\tilde{B}}_{i}$, $i\in L_{n}$, and return $Z_{L_{n}}$ to the user. We distribute the encoded matrices to the computing nodes in the following way. Let $J={\left[\begin{array}{ccc} J_{1} & J_{2} & \cdots J_{N} \end{array}\right]}^{T}$ be the distribution vector where $J_{n}\in \left[0:N_{1}\right]$ is the number of distributed encoded matrices given to the *n*-th computing node. Then, we have $\left|L_{n}\right|=J_{n},1^{T}J=N_{1}$. It has been proved in [19] that when

(10) $$\begin{array}{c} B_{P}^{T}J\leq \left(l_{\Delta }s\right)1_{M}, \end{array}$$

the security constraint (2) is satisfied. The physical meaning of (10) is that the number of encoded matrices for computing nodes in every colluding set must be smaller than the minimal number of random matrices appended in $A^{*}$ or $B^{*}$, which is $l_{\Delta }s$. Furthermore, the decodability constraint (3) is guaranteed by the following inequality [19]:

(11) $$\begin{array}{c} 1^{T}J=N_{1}\geq l_{\Delta }\left(sd+2s\right)+ts\left(d+1\right)-1. \end{array}$$

It means that the encoded copies $N_{1}$ must be no smaller than $l_{\Delta }\left(sd+2s\right)+ts\left(d+1\right)-1$ for decoding the desired results C=AB without error.

### 2.2. Storage, Communication and Computing Requirements of Each Computing Node

The amount of storage each encoded copy $\left({\tilde{A}}_{i},{\tilde{B}}_{i}\right)$ occupies is $t_{0}s_{0}+s_{0}d_{0}$. Suppose computing node *n*’s storage capacity is $M_{n}$, then, if $t_{0}s_{0}+s_{0}d_{0}+t_{0}d_{0}>M_{n}$, computing node *n* can not even store one encoded copy of $\left({\tilde{A}}_{i},{\tilde{B}}_{i}\right)$ and its corresponding answer, i.e., $Z_{i}={\tilde{A}}_{i}{\tilde{B}}_{i}$. If 

(12) $$\begin{array}{c} M_{n}\geq t_{0}s_{0}+s_{0}d_{0}+t_{0}d_{0}, \end{array}$$

then the computing node could store one encoded copy $\left({\tilde{A}}_{i},{\tilde{B}}_{i}\right)$, $i\in L_{n}$, compute the multiplication $Z_{i}={\tilde{A}}_{i}{\tilde{B}}_{i}$, return the corresponding result and then retrieve another encoded copies from the user for further computation. Hence, (12) must be satisfied for all n∈[1:N]. Written in vector form, we have

(13) $$\begin{array}{c} \left(t_{0}s_{0}+s_{0}d_{0}+t_{0}d_{0}\right)1_{N}\leq M. \end{array}$$

Suppose computing node *n*’s computation speed is $V_{n}$ multiplications per second, then the time it takes for the user to complete the computation assigned to it, is

$$\begin{array}{c} Q_{n}^{C}=\frac{J_{n}}{V_{n}}t_{0}s_{0}d_{0}. \end{array}$$

Further suppose that the uplink and downlink capacity between the user and computing node *n* are $C_{n}^{U}$ and $C_{n}^{D}$ symbols per second, respectively. Then, the amount of upload delay incurred at computing node *n* is

$$\begin{array}{c} Q_{n}^{U}=\frac{J_{n}}{C_{n}^{U}}\left(t_{0}s_{0}+s_{0}d_{0}\right), \end{array}$$

and the amount of download delay incurred at computing node *n* is

$$\begin{array}{c} Q_{n}^{D}=\frac{J_{n}}{C_{n}^{D}}t_{0}d_{0}. \end{array}$$

Then the total amount of delay incurred at computing node *n* when assigned with $J_{n}$ number of encoded copies is

(14) $$\begin{array}{c} Q_{n}=Q_{n}^{U}+Q_{n}^{D}+Q_{n}^{C}, \end{array}$$

where we have assumed that the computing nodes can only do one of the three actions at any time instant: compute or receive upload or send download. This is also in line with the assumption that the computing nodes may not have enough memory to store all $J_{n}$ copies all at once. Rather, it receives one copy, computes, and then sends it back to the user and then retrieves the next copy and repeats.

Thus, the total delay incurred for this computation is

(15) $$\begin{array}{c} Q=\max_{n\in [1:N]}Q_{n}=\max_{n\in [1:N]}\left\{\frac{J_{n}}{V_{n}}t_{0}s_{0}d_{0}+\frac{J_{n}}{C_{n}^{U}}\left(t_{0}s_{0}+s_{0}d_{0}\right)+\frac{J_{n}}{C_{n}^{D}}t_{0}d_{0}\right\}, \end{array}$$

and we require that the total delay is no larger than a given threshold $Q_{\mathrm{th}}$, i.e.,

(16) $$\begin{array}{c} \max_{n\in [1:N]}\left\{\frac{J_{n}}{V_{n}}t_{0}s_{0}d_{0}+\frac{J_{n}}{C_{n}^{U}}\left(t_{0}s_{0}+s_{0}d_{0}\right)+\frac{J_{n}}{C_{n}^{D}}t_{0}d_{0}\right\}\leq Q_{\mathrm{th}}. \end{array}$$

Besides the delay constraint, cost should also be considered for efficient SDMM. More specifically, the cost we consider is comprised of the computation cost of computing nodes and the data transmission cost, where the data transmission cost can be can be written twice divided into the upload and download transmission cost. More specifically, we assume that the upload and download transmission cost for computing node *n* is $c_{n}^{U}$, and $c_{n}^{D}$ per symbol, and the computation cost of each multiplication at computing node *n* is $c_{n}^{C}$, then the total required cost for the user doing the secure matrix multiplication of matrices A and B is

(17) $$\begin{array}{c} U=U_{U}+U_{D}+U_{C}, \end{array}$$

where $U_{U}$ is the upload cost, which is given by

$$\begin{array}{c} U_{U}=\left(t_{0}s_{0}+s_{0}d_{0}\right)\sum_{n=1}^{N}J_{n}c_{n}^{U}, \end{array}$$

$U_{D}$ is the download cost, which is given by

$$\begin{array}{c} U_{D}=t_{0}d_{0}\sum_{n=1}^{N}J_{n}c_{n}^{D}, \end{array}$$

and $U_{C}$ is the computation cost, which is given by

$$\begin{array}{c} U_{C}=t_{0}s_{0}d_{0}\sum_{n=1}^{N}J_{n}c_{n}^{C}. \end{array}$$

### 2.3. Problem Formulation

In this work, we would like to jointly optimize the distribution vector J, and the matrix split parameter $\left(t,s,d,l_{\Delta }\right)$ such that the cost of the user, defined in (17), is minimized. At the same time, the security constraint (10), the decodability constraint (11), the storage constraint (13), and the delay constraint (16) must be satisfied.

## 3. The Feasible Set of (T,S,D)

Since we are splitting the two matrices A and B as shown in (4), it is natural to assume that *t*, *s*, and *d* have to take values such that *T*, *S*, and *D* be divisible by *t*, *s*, and *d*, respectively. For example, if T=5, *t* can only take values in the set {1,5}, because T=5 is not divisible by 2,3,4. However, this significantly limit the values that (t,s,d) can take and may provide a high cost for the user.

In this section, we propose a better and more general way as follows: we allow any t,s,d values, and to make the matrix splittable, we append zeros to the original matrix, i.e., append $\bar{s}$ columns and $\bar{t}$ rows to the matrix A and append $\bar{s}$ rows and $\bar{d}$ columns to the matrix B, such that $(T+\bar{t})/t$, $(S+\bar{s})/s$, and $(D+\bar{d})/d$ are integers. This increases the dimension of the two matrices but enables us to split them into blocks in a more flexible way. For example, $A\in F^{5\times 4}$, i.e., T=5,S=4, and we would like to take t=2,s=2. However, T=5 is not divisible by t=2. Then, we can append one row of zeros to A so that the appended matrix has dimension 6×4 and thus can be divisible by t=2,s=2.

More generally, we propose that for any (t,s,d) with t∈[1:T],s∈[1:S],d∈[1:D], we may append (Tmodt) many rows to the bottom of matrix A and (Smods) many columns to the right side of matrix A. Similarly, we append (Smods) many rows to the bottom of matrix B and (Dmodd) many columns to the right side of matrix B. As a result, instead of (5), we have

(18) $$\begin{array}{c} t_{0}=\left\lceil \frac{T}{t}\right\rceil ,\;s_{0}=\left\lceil \frac{S}{s}\right\rceil ,\;d_{0}=\left\lceil \frac{D}{d}\right\rceil . \end{array}$$

As can be seen, not padding zeros and only using (t,s,d) that is a divisor of (T,S,D) is a special case.

Since we are considering padding zeros, it is also possible to have $t\in \left\{T+1,T+2,\cdots \right\}$ or $s\in \left\{S+1,S+2,\cdots \right\}$ or $d\in \left\{D+1,D+2,\cdots \right\}$. We show in the next lemma that this will only increase the cost at the user, defined in (17), for $t\in \left\{T+1,T+2,\cdots \right\}$ and $d\in \left\{D+1,D+2,\cdots \right\}$.

**Lemma 1.**  *To minimize the cost at the user, i.e., (17), it is sufficient to consider t∈[1:T] and d∈[1:D].*

**Proof.**  For $t\in \left\{T+1,T+2,\cdots \right\}$ and $d\in \left\{D+1,D+2,\cdots \right\}$, the only decodability constraint (11) becomes relaxed, and other constraints are unchanged. In this case, we can prove that the optimal cost will increase compared to t∈[1:T] and d∈[1:D]. Please refer to Appendix A for detailed proof.    □

**Remark 1.**  *The case of s is different from the cases of t and d. When $\left(l_{\Delta },t,d\right)$ is fixed, from security constraint (10) and decodability constraint (11), we see that on one hand, increasing s increases the number of blocks, but on the other hand, it also relaxes the security constraint. When computing nodes are heterogeneous, i.e., computing nodes have different computation cost, upload transmission cost and download transmission cost, the increase in s does not necessarily increase the total cost, because due to the more relaxed security constraint, we can distribute more blocks to computing nodes with lower costs. As a result, when we apply the strategy of appending zeros, the optimal s may not take values in [1:S].*

After the above discussions, the problem described in Section 2.3 can be formally formulated as

(19a)minJ,t,s,d,lΔJTcUt0s0+s0d0+JTcDt0d0+JTcCt0s0d0(19b)s.t.(10),(11),(19c)t0s0+s0d0+t0d01N≤M,(19d)maxn∈[1:N]JnVnt0s0d0+JnCnUt0s0+s0d0+JnCnDt0d0≤Qth,(19e)t0=Tt,s0=Ss,d0=Dd(19f)1≤t≤T,1≤s,1≤d≤D,lΔ≥1,(19g)t,s,d,lΔ∈Z+,J∈NN×1.

where (19b) provides the security constraint and decodability constraint, (19c) is the storage constraint with *N* computing nodes’ storage capacity vector defined as $M={\left[\begin{array}{ccc} M_{1} & \cdots & M_{N} \end{array}\right]}^{T}$, and (19d) is the delay constraint. In the cost function (19a), we have defined the upload transmission cost vector, the download transmission cost vector and the computation cost vector of *N* computing nodes as $c^{U}={\left[c_{1}^{U},c_{2}^{U},\cdots ,c_{N}^{U}\right]}^{T}\in R^{N\times 1}$, $c^{D}={\left[c_{1}^{D},c_{2}^{D},\cdots ,c_{N}^{D}\right]}^{T}\in R^{N\times 1}$ and $c^{C}={\left[c_{1}^{C},c_{2}^{C},\cdots ,c_{N}^{C}\right]}^{T}\in R^{N\times 1}$, respectively. Note that the scheme of appending zeros makes the dimension of every block in A and B to be $t_{0}\times s_{0}=\left\lceil \frac{T}{t}\right\rceil \times \left\lceil \frac{S}{s}\right\rceil$ and $s_{0}\times d_{0}=\left\lceil \frac{S}{s}\right\rceil \times \left\lceil \frac{D}{d}\right\rceil$, respectively, as indicated by (19e). Furthermore, note that in (19f), while the values of *t* and *d* are limited to [1:T] and [1:D], respectively, the value of *s* does not have an upper bound due to Remark 1.

## 4. Algorithm Design

Due to coupling variables, integer constraints and nonlinear constraints and objective function of the problem in (19), it is hard to find a global optimal or suboptimal solution. In the following, we propose an algorithm to obtain a feasible solution.

Coupling variables in Problem (19) inspires us to utilize the alternating optimization (AO) technique. Then, a feasible solution to Problem (19) can be obtained by solving the next two subproblems: one is fixing (t,s,d) to optimize $\left(J,l_{\Delta }\right)$, and the other is optimizing (t,s,d) given $\left(J,l_{\Delta }\right)$.

### 4.1. Optimization Subproblem of $\left(J,l_{\Delta }\right)$ for a Fixed (T,S,D)

In this subsection, for a fixed (t,s,d), the optimization subproblem of Problem (19) corresponding to $\left(J,l_{\Delta }\right)$ is given as

(20a)minJ,lΔ(19a)(20b)s.t.(10),(11),(20c)Jn≤Qth1Vnt0s0d0+1CnUt0s0+s0d0+1CnDt0d0,∀n∈[1:N](20d)lΔ≥1,lΔ∈Z+,J∈NN×1.

Note that when (t,s,d) is fixed, the corresponding $\left(t_{0},s_{0},d_{0}\right)$ is also fixed according to (19e). Further note that when (t,s,d) is fixed, the objective function (20a) is only a function of J, and not $l_{\Delta }$. Due to the fact that $J_{n},c_{n}^{U},c_{n}^{D},c_{n}^{C}\geq 0,n=1,\ldots ,N$, the inequality of (11) must be satisfied with the equality when $J^{*}$ is optimal. Hence, (11) can be rewritten as follows

(21) $$\begin{array}{c} 1^{T}J=l_{\Delta }\left(sd+2s\right)+ts\left(d+1\right)-1. \end{array}$$

With equality (21), $l_{\Delta }$ can be expressed as a function of J, i.e., $l_{\Delta }=\frac{1^{T}J-ts\left(d+1\right)+1}{sd+2s}$. Then, substituting $l_{\Delta }$ in Problem (20) as a the function of J, Problem (20) can be reformulated as

(22a)minJ(19a)(22b)s.t.(20c),(22c)BPTJ≤(1TJ−ts(d+1)+1d+2)1M,(22d)1TJ−ts(d+1)+1sd+2s≥1,1TJ−ts(d+1)+1sd+2s∈Z+,J∈NN×1.

Problem (22) is an integer linear programming problem with only one optimizing variable J. This problem can be solved using MATLAB function “intlinprog”. MATLAB’s built-in “intlinprog” function is based on the branch and bound (BnB) algorithm and the interior point method [20,21] and is typically used to solve integer linear programming problems, such as the one in (22).

For certain system parameters and (t,s,d) values, Problem (22) is not feasible. To identify a necessary condition for the feasibility of Problem (22), we have the following lemma.

Before presenting the lemma, we define a variable *p* as the smallest number of colluding sets that contain all computing nodes. For example, for the collusion pattern represented by incidence matrix (1), *p* is equal to 3, because three colluding sets, i.e., {1,2,3},{1,4},{5}, include all computing nodes, and any 2 colluding sets in the collusion pattern can not include all computing nodes.

**Lemma 2.**  *For fixed parameters (t,s,d), if Problem (20) is feasible, the following inequalities must be satisfied:*

$$\begin{array}{cc} \left\lceil \frac{tsd+ts-1}{s(p-d-2)}\right\rceil & \leq \frac{Y-tsd-ts+1}{sd+2s}\;\;\mathrm{and}\;\;p-d-2>0 \end{array}$$

*where Y is defined as*

(23) $$\begin{array}{c} Y≜\sum_{n=1}^{N}\frac{Q_{\mathrm{th}}}{\frac{1}{V_{n}}t_{0}s_{0}d_{0}+\frac{1}{C_{n}^{U}}\left(t_{0}s_{0}+s_{0}d_{0}\right)+\frac{1}{C_{n}^{D}}t_{0}d_{0}}, \end{array}$$

*where $\left(t_{0},s_{0},d_{0}\right)$ satisfies (19e). Variable p is defined as the smallest number of colluding sets that contain all computing nodes.*

**Proof.**  Let us first derive a lower bound of $l_{\Delta }$. According to the second assumption made about the collusion pattern in Section 2, every computing node must appear in at least one colluding set. So, we have

(24) $$\begin{array}{c} \sum_{n=1}^{N}J_{n}\leq \sum_{m\in P^{\prime }}\sum_{n\in T_{m}}J_{n} \end{array}$$

for any $P^{\prime }$ which is a subset of colluding sets in P that include all computing nodes, i.e., $\left\{i\right\}\in T_{j}$ for some $T_{j}\in P^{\prime }$ for any i=1,2,⋯,N. For example, in the collusion pattern represented by the incidence matrix in (1), $P^{\prime }$ may be {{1,2,3},{1,4},{5}}, {{1,2,3},{2,4},{5}}, {{1,2,3},{3,4},{5}}, or {{1,4},{2,4},{3,4},{5}}.

The constraint (10) can be rewritten as

(25) $$\begin{array}{c} \sum_{n\in T_{m}}J_{n}\leq l_{\Delta }s, \end{array}$$

where $T_{m}$ is the *m*-th colluding set. Inequality (25) shows that the total number of encoded matrices received by computing nodes in every colluding set can not be more than that of random matrices. Hence, from (25), we have

(26) $$\begin{array}{c} \sum_{m\in P^{\prime }}\sum_{n\in T_{m}}J_{n}\leq \sum_{m\in P^{\prime }}l_{\Delta }s\leq pl_{\Delta }s \end{array}$$

Thus, from (21), (24), and (26), we have

$$\begin{array}{c} l_{\Delta }\left(sd+2s\right)+ts\left(d+1\right)-1=\sum_{n=1}^{N}J_{n}\leq pl_{\Delta }s. \end{array}$$

When p−d−2>0 is satisfied, $l_{\Delta }$ must satisfy

(27) $$\begin{array}{c} l_{\Delta }\geq \left\lceil \frac{tsd+ts-1}{s(p-d-2)}\right\rceil . \end{array}$$

On the other hand, when p−d−2>0 is not satisfied, there exists no feasible $l_{\Delta }$.

Next, we derive an upper bound on $l_{\Delta }$. We have

(28) $$\begin{array}{cc} \; & l_{\Delta }\left(sd+2s\right)+ts\left(d+1\right)-1=\sum_{n=1}^{N}J_{n} \end{array}$$

(29) $$\begin{array}{cc} \; & \leq \sum_{n=1}^{N}\frac{Q_{\mathrm{th}}}{\frac{1}{V_{n}}t_{0}s_{0}d_{0}+\frac{1}{C_{n}^{U}}\left(t_{0}s_{0}+s_{0}d_{0}\right)+\frac{1}{C_{n}^{D}}t_{0}d_{0}}, \end{array}$$

where (28) follows from (21), and (29) follows from (20c). Hence, an upper bound on $l_{\Delta }$ is given by

(30) $$\begin{array}{c} l_{\Delta }\leq \frac{Y-tsd-ts+1}{sd+2s}, \end{array}$$

where *Y* is as defined in (23).

If Problem (20) is feasible, we must have that p−d−2>0, and the upper bound of $l_{\Delta }$ in (30) must be greater than or equal to the lower bound of $l_{\Delta }$ in (27).

Hence, the proof is complete.    □

Based on Lemma 2, Algorithm 1 is proposed to solve Problem (20), where we check the necessary conditions of the feasibility of Problem (20) before solving it using the MATLAB “intlinprog” function.
**Algorithm 1** Iterative Algorithm for Problem (20)**Input:** (t,s,d), and $\left(t_{0},s_{0},d_{0}\right)$ calculated according to (19e)**Output:** $J,l_{\Delta }$.  1: **if**$\frac{Y-tsd-ts+1}{sd+2s}\geq \left\lceil \frac{tsd+ts-1}{s(p-d-2)}\right\rceil$ and p−d−2>0 **then**  2:    Solve Problem (22) with MATLAB function “intlinprog”.  3: **else**  4:    Problem (20) is infeasible.  5: **end if**

### 4.2. Optimization Subproblem of (T,S,D) for a Fixed $\left(J,l_{\Delta }\right)$

In this subsection, given $\left(J,l_{\Delta }\right)$, the optimization subproblem of Problem (19) corresponding to (t,s,d) is formulated as

(31a)mint,s,d(19a)(31b)s.t.(10),(11),(19c),(19d),(19e)(31c)1≤t≤T,1≤s,1≤d≤D,(31d)d≤p−3,(31e)t,s,d∈Z+,

where constraint (31d) is derived from Lemma 2.

Ceiling functions, i.e., $\left\lceil \frac{T}{t}\right\rceil ,\left\lceil \frac{S}{s}\right\rceil ,\left\lceil \frac{D}{d}\right\rceil$ in (19e), and integer constraint (31e) make Problem (31) hard to address. We can solve this subproblem by relaxing the ceiling functions, i.e., Problem (31) can be recast as

(32a)mint,s,dJTcUTSt−1s−1+SDs−1d−1+JTcDTDt−1d−1+JTcCTSDt−1s−1d−1(32b)s.t.BPTJlΔs−1≤1M,(32c)lΔ(sd+2s)+ts(d+1)−11TJ≤1,(32d)TSt−1s−1+SDs−1d−1+TDt−1d−11N≤M,(32e)JnVnTSDt−1s−1d−1+JnCnUTSt−1s−1+SDs−1d−1+JnCnDTDt−1d−1≤Qth,∀n∈[1:N](32f)(31c),(31d),(31e).

Problem (32) is an integer geometric programming problem which can be solved using the Matlab toolbox YALMIP directly. MATLAB’s built-in “YALMIP” function is based on the interior point method and BnB algorithm [22,23] and is typically used to solve integer geometric programming problems, such as the one in (32).

### 4.3. The Proposed Alternating Optimization (AO) Algorithm

Based on the above discussions of the two subproblems, we propose an AO algorithm as follows: In every AO iteration, for a fixed $\left(t,s,d\right)=\left(t^{\left(\tau \right)},s^{\left(\tau \right)},d^{\left(\tau \right)}\right)$, and $\left(t_{0},s_{0},d_{0}\right)$, which is calculated as $\left(t_{0},s_{0},d_{0}\right)=\left(\left\lceil \frac{T}{t^{\left(\tau \right)}}\right\rceil ,\left\lceil \frac{S}{s^{\left(\tau \right)}}\right\rceil ,\left\lceil \frac{D}{d^{\left(\tau \right)}}\right\rceil \right)$, we use Algorithm 1 to solve (22). Then, for the output $\left(J,l_{\Delta }\right)$ of Algorithm 1, we solve Problem (32) with YALMIP directly and obtain $\left(t^{\left(\tau +1\right)},s^{\left(\tau +1\right)},d^{\left(\tau +1\right)}\right)$.

Since Problem (32) is obtained by the relaxation of the ceiling functions, we may face the problem where even though the (t,s,d) found by YALMIP are integers, which we call $\left(\bar{t},\bar{s},\bar{d}\right)$, the corresponding $\frac{T}{\bar{t}},\frac{S}{\bar{s}}$, and $\frac{D}{\bar{d}}$ in Problem (32) may not be integers. In order to overcome this problem, for the converged solution $\left(t^{*},s^{*},d^{*}\right)$ of the AO, we check whether constraints (19c) and (19d) in the original problem (19) are satisfied according to the definition of (19e). If they are, $\left(t^{*},s^{*},d^{*}\right)$ is taken as the solution to Problem (31), and the corresponding block dimensions $\left(t_{0},s_{0},d_{0}\right)$ are taken to be $\left(\left\lceil \frac{T}{t^{*}}\right\rceil ,\left\lceil \frac{S}{s^{*}}\right\rceil ,\left\lceil \frac{D}{d^{*}}\right\rceil \right)$ by padding zeros. If they are not, then we can employ an exhaustive search within a neighborhood near the converged solution $\left(t^{*},s^{*},d^{*}\right)$ for a feasible solution or restart the algorithm with a new random initial point $\left(t^{\left(0\right)},s^{\left(0\right)},d^{\left(0\right)}\right)$. In a time-constrained system, we can also abandon optimizing (t,s,d) and simply use the initial values $\left(t^{\left(0\right)},s^{\left(0\right)},d^{\left(0\right)}\right)$ to obtain a timely solution.

Finally, the proposed AO algorithm to solve Problem (19) is summarized in Algorithm 2. (The source code can be found in the following link: https://github.com/SendBullet/SDMM-opt (accessed on 14 March 2024))
**Algorithm 2** Alternating Optimization Algorithm for Problem (19)  1:Initialize $t^{\left(0\right)},s^{\left(0\right)},d^{\left(0\right)},\tau =0$ and the tolerance ϵ, where $t^{\left(0\right)},s^{\left(0\right)},d^{\left(0\right)}$ are chosen from divisors of (T,S,D) randomly.  2:**repeat**  3:   Given $\left(t,s,d\right)=\left(t^{\left(\tau \right)},s^{\left(\tau \right)},d^{\left(\tau \right)}\right)$, calculate $J^{\left(\tau +1\right)},l_{\Delta }^{\left(\tau +1\right)}$ by Algorithm 1.  4:   Given $J=J^{\left(\tau +1\right)},l_{\Delta }=l_{\Delta }^{\left(\tau +1\right)}$, calculate $\left(t^{\left(\tau +1\right)},s^{\left(\tau +1\right)},d^{\left(\tau +1\right)}\right)$ by solving Problem (32) with YALMIP directly.  5:   Set τ=τ+1.  6:**until** The fractional increase of the objective function of Problem (19) is less than ϵ.  7:**if** Constraints (19c), (19d) and (19e) are satisfied simultaneously **then**  8:   Output $J^{\left(\tau \right)},l_{\Delta }^{\left(\tau \right)},\left(t^{\left(\tau \right)},s^{\left(\tau \right)},d^{\left(\tau \right)}\right)$ and block dimension $\left(t_{0},s_{0},d_{0}\right)=\left(\left\lceil \frac{T}{t^{\left(\tau \right)}}\right\rceil ,\left\lceil \frac{S}{s^{\left(\tau \right)}}\right\rceil ,\left\lceil \frac{D}{d^{\left(\tau \right)}}\right\rceil \right)$.  9:**else**10:   Return to step 1 and restart with a new random initial point.11:**end if**

### 4.4. Complexity Analysis

The complexity of Algorithm 2 per iteration mainly lies in Steps 3 and 4. In Step 3, the complexity of Algorithm 1 is derived from solving Problem (20) by MATLAB function “intlinprog” which uses BnB method. By omitting the lower-order terms, the main complexity of Algorithm 1 per iteration is $O(2^{N})$, where *N* is the number of computing nodes. In Step 4, similarly, the main complexity of solving Problem (32) by YALMIP with BnB method is $O(2^{3})$, where 3 is the dimension of optimizing variables (t,s,d) [24]. Hence, by neglecting the lower-order terms, the approximate computational complexity of Algorithm 2 per iteration is $O(2^{N})$ when N≥3, and $O(2^{3})$ when N≤2. As can be seen, the complexity scales exponentially with the number of computing nodes.

## 5. Simulation Results

In this section, we provide simulation results to evaluate the performance of the two-sided SDMM under arbitrary collusion pattern. We consider two collusion patterns. The first one has N=11 computing nodes with the collusion pattern being $P_{1}=\{\left\{1,4\right\},\left\{2,5\right\},\left\{1,2,6\right\},\left\{3,7\right\},$ {4,5,6,7},{8},{9},{10},{11}}, while the second one consists of N=20 computing nodes with the collusion pattern being $P_{2}=\{\left\{1,2,3,4,5,6\right\},$ {6,7,8,9,10,11},{12,13},{14},{13,15},{16,17},{18},{19},{20}} So, for these two collusion patterns, the smallest number of colluding sets containing all of the computing nodes is p=7 and p=9, respectively. The stopping criterion in Algorithms 2 is set to $\epsilon =10^{-8}$ [25]. Other system parameters are listed in the Table 1.

For simplicity, our proposed scheme in this paper is denoted by “Pro.”. Then, the following two benchmarks are considered to compare with our proposed scheme:

(1) “N/0.”: In this scenario, we do not append zeros to the input matrices. The optimization subproblem corresponding to (t,s,d) for a fixed $\left(J,l_{\Delta }\right)$ is solved by exhaustive search in feasible pairs $\left(\tilde{t},\tilde{s},\tilde{d}\right)$, which are divisors of (T,S,D). Other details are similar to Algorithm 2. This corresponds to the optimal performance of AO when no zeros are appended.
(2) “SE.”: First, (t,s,d) is initialized by divisors of (T,S,D) randomly. Then, we solve Problem (22) to obtain $\left(J,l_{\Delta }\right)$. This is a low complexity algorithm where no zeros are appended, and also, (t,s,d) are randomly chosen without being optimized. Only $\left(J,l_{\Delta }\right)$ are optimized for the fixed randomly chosen (t,s,d).

**Figure 2:**
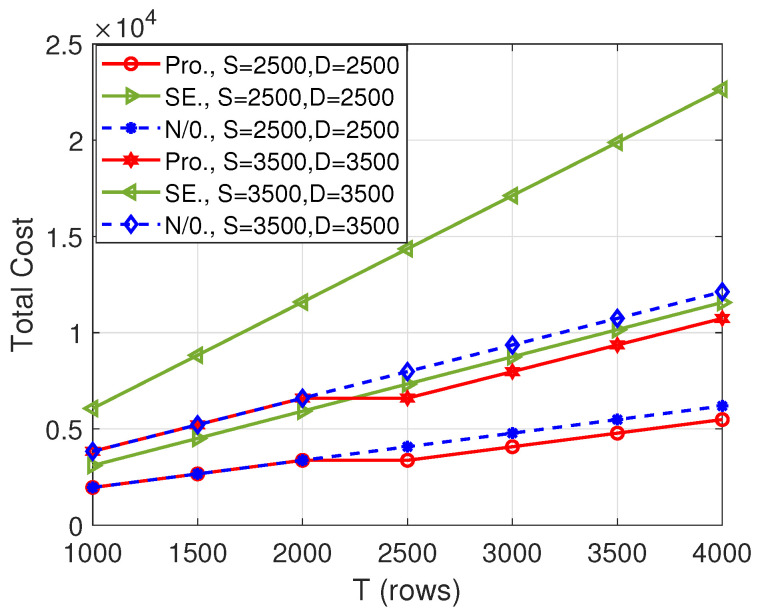
Total cost versus *T* when N=11.

First, we consider the collusion pattern $P_{1}$ and the number of computing nodes being 11. Figure 2 shows the total cost versus the number of rows of matrix A, i.e., *T*. Firstly, with the increase in *T*, the total cost of all schemes increases, which is caused by the growth of input matrix dimension. Secondly, our proposed algorithm outperforms the “N/0.” scheme when T≥2500. This means that when T≥2500, it is better to append zeros to the matrices to obtain a lower cost. Thirdly, our proposed algorithm always performs better than the “SE” scheme, which demonstrates the necessity of both appending zeros and performing AO. Lastly, from the comparison between (S,D)=(2500,2500) and (S,D)=(3500,3500), we can observe that the cost of the proposed scheme increases with the increase of the size of the matrices.

**Figure 3:**
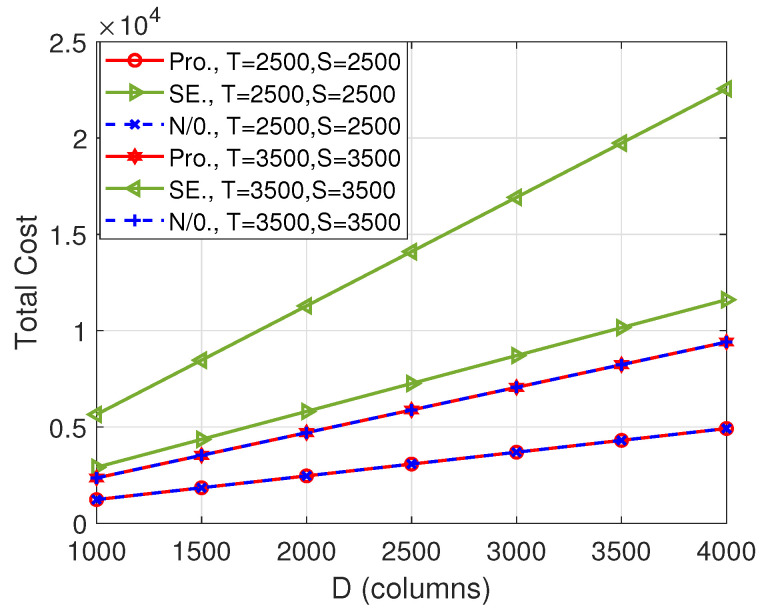
Total cost versus *D* when N=11.

Figure 3 plots the total cost versus the number of columns of matrix B, i.e., *D*. Similar to Figure 2, the difference between our proposed scheme and the “SE” scheme becomes larger with the increase in the dimensions of the input matrices. However, the “N/0.” scheme achieves the same total cost as our proposed algorithm. This shows that, in this case, there is no need to pad zeros. Though the proposed scheme and the “N/0.” scheme have the same performance, the proposed scheme has less complexity because it can avoid the exhaustive search of the “N/0.” scheme.

**Figure 4:**
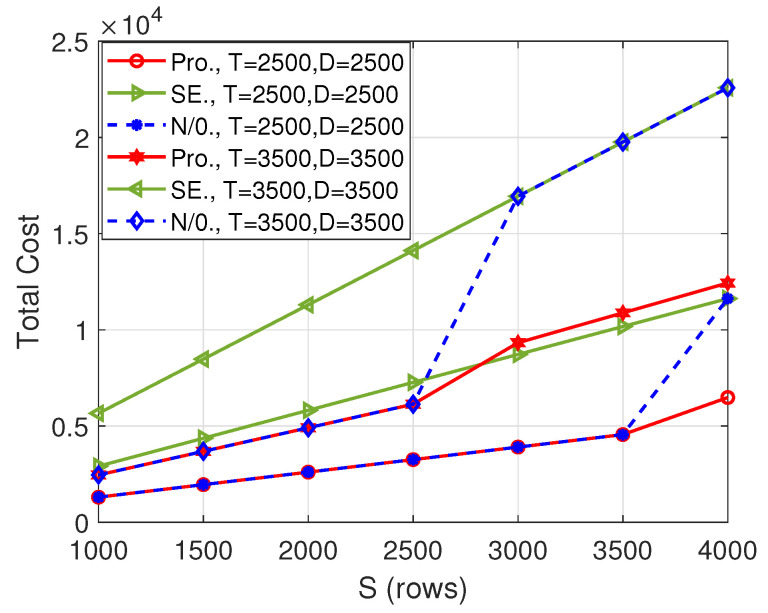
Total cost versus *S* when N=11.

Figure 4 illustrates the total cost versus the number of columns of matrix A, i.e., *S*, which is also the number of rows of the matrix B. Although the total cost of our proposed scheme is the same as that of the “N/0.” scheme for some *S* values, the gain of our proposed algorithm over the “N/0.” scheme increases with the increase in *S*. In fact, the gain is very significant for large *S* values, for example, when S=4000, the total cost incurred by the proposed scheme is only 55.77% of the “N/0.” scheme when (T,D)=(2500,2500) and 55.07% when (T,D)=(3500,3500).

**Figure 5:**
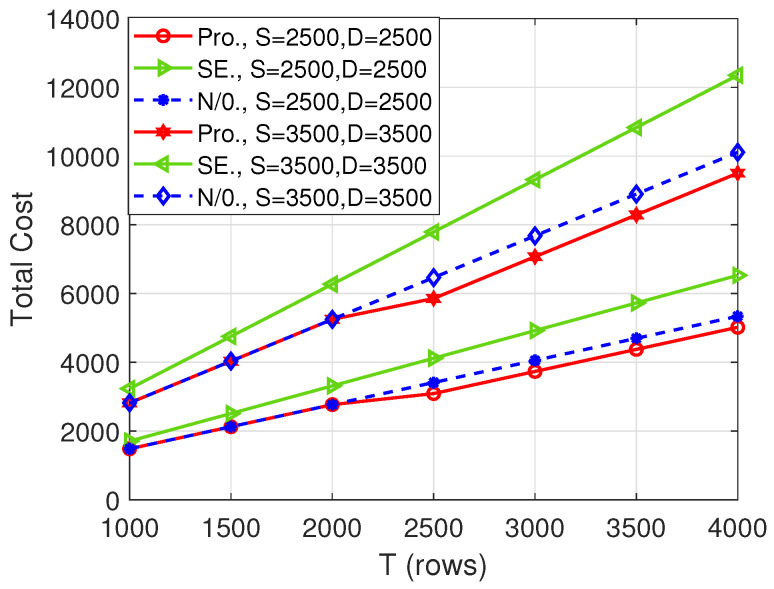
Total cost versus *T* when N=20.

**Figure 6:**
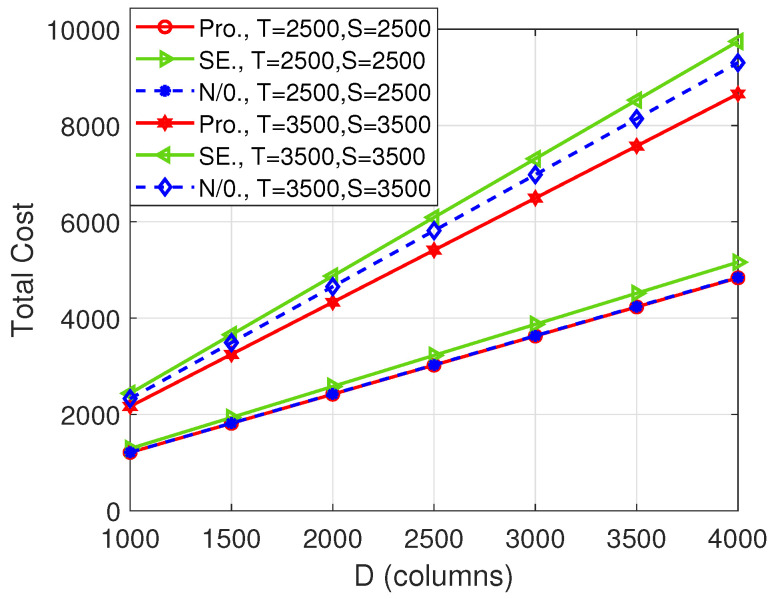
Total cost versus *D* when N=20.

**Figure 7:**
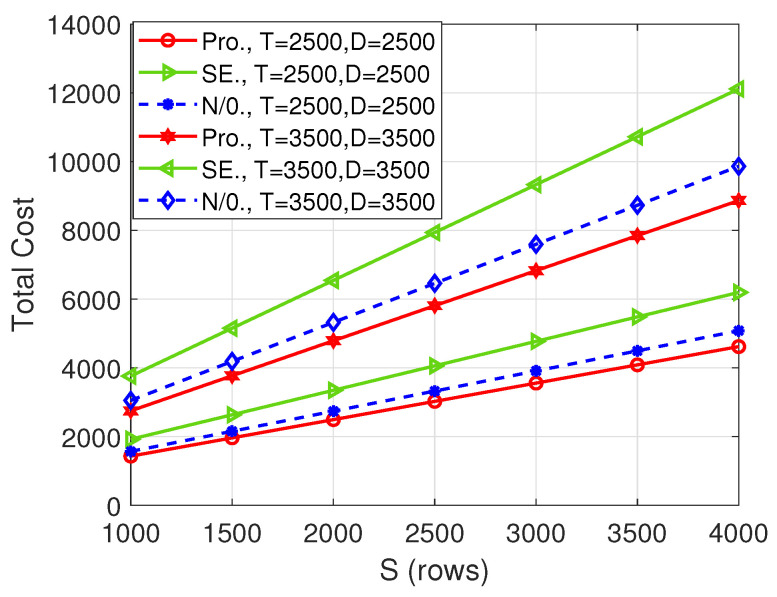
Total cost versus *S* when N=20.

Figure 5, Figure 6 and Figure 7, respectively, depict the total cost with respect to *T*, *S*, and *D* when the number of computing nodes is 20. Similarly, our proposed scheme strictly outperforms the other two benchmarks in some cases, which further shows the superiority of the proposed scheme. Comparing Figure 2, Figure 3 and Figure 4 with Figure 5, Figure 6 and Figure 7, respectively, we see that the total cost decreases significantly with the increase in the number of computing nodes. Thus, when possible, the user should utilize more computing nodes to reduce the total cost.

## 6. Conclusions

In this paper, we investigated the minimization problem of the total cost, comprised of the computation cost and the communication cost, in the system of two-sided SDMM under an arbitrary collusion pattern. For realizing SDMM, we split the two input matrices into many blocks and appended some extra blocks of random matrices to guarantee the security of the two input matrices. Then, the matrix multiplication is calculated based on the encoded copies in the computing nodes. Our aim is to minimize the total cost, while ensuring the security constraint of the two input matrices, the decodability constraint of the desired result of the multiplication, the storage capacity of the computing nodes, and the delay constraint. The distribution vector, the number of appended random matrices, and all matrix splitting factors were optimized. In order to overcome divisibility problem of matrix splitting, we firstly proposed a strategy of appending zeros to the two input matrices and then discussed the value ranges of some matrix splitting factors for the optimality of the problem. Next, an AO algorithm was provided to obtain a feasible solution. Furthermore, to verify the feasibility of the proposed optimization problem, some necessary conditions were provided. Numerical results demonstrated that our proposed scheme achieves a lower total cost compared to the scheme without appending zeros and the scheme without AO optimization.

## Figures and Tables

**Table 1:** System parameters.

Parameters	Values for N=11	Values for N=20
Storage capacity M	$M_{1}=M_{2}=M_{3}=M_{4}=1\times 10^{6}$, $M_{5}=M_{6}=M_{7}=M_{8}=2\times 10^{6}$, $M_{9}=M_{10}=M_{11}=3\times 10^{6}$	$M_{1}=\cdots =M_{7}=1\times 10^{6}$, $M_{8}=\cdots =M_{15}=2\times 10^{6}$, $M_{16}=\cdots =M_{20}=3\times 10^{6}$
Computation speed $V_{n}$	$V_{1}=V_{2}=V_{3}=V_{4}=2\times 10^{8}$, $V_{5}=V_{6}=V_{7}=V_{8}=5\times 10^{8}$, $V_{9}=V_{10}=V_{11}=8\times 10^{8}$	$V_{1}=\cdots =V_{7}=2\times 10^{8}$, $V_{8}=\cdots =V_{15}=5\times 10^{8}$, $V_{16}=\cdots =V_{20}=8\times 10^{8}$
Uplink Capacity $C_{n}^{U}$	$C_{1}^{U}=C_{2}^{U}=C_{3}^{U}=C_{4}^{U}=1024\times 10^{4}$, $C_{5}^{U}=C_{6}^{U}=C_{7}^{U}=C_{8}^{U}=2048\times 10^{4}$, $C_{9}^{U}=C_{10}^{U}=C_{11}^{U}=4096\times 10^{4}$	$C_{1}^{U}=\cdots =C_{7}^{U}=1024\times 10^{4}$, $C_{8}^{U}=\cdots =C_{15}^{U}=2048\times 10^{4}$, $C_{16}^{U}=\cdots =C_{20}^{U}=4096\times 10^{4}$
Downlink Capacity $C_{n}^{D}$	$C_{1}^{D}=C_{2}^{D}=C_{3}^{D}=C_{4}^{D}=1024\times 10^{4}$, $C_{5}^{D}=C_{6}^{D}=C_{7}^{D}=C_{8}^{D}=2048\times 10^{4}$, $C_{9}^{D}=C_{10}^{D}=C_{11}^{D}=4096\times 10^{4}$	$C_{1}^{D}=\cdots =C_{7}^{D}=1024\times 10^{4}$, $C_{8}^{D}=\cdots =C_{15}^{D}=2048\times 10^{4}$, $C_{16}^{D}=\cdots =C_{20}^{D}=4096\times 10^{4}$
Upload cost $c^{U}$	$c_{1}^{U}=c_{2}^{U}=c_{3}^{U}=c_{4}^{U}=3\times 10^{-8}$, $c_{5}^{U}=c_{6}^{U}=c_{7}^{U}=c_{8}^{U}=4\times 10^{-8}$, $c_{9}^{U}=c_{10}^{U}=c_{11}^{U}=5\times 10^{-8}$	$c_{1}^{U}=\cdots =c_{7}^{U}=3\times 10^{-8}$, $c_{8}^{U}=\cdots =c_{15}^{U}=4\times 10^{-8}$, $c_{16}^{U}=\cdots =c_{20}^{U}=5\times 10^{-8}$
Download cost $c^{D}$	$c_{1}^{D}=c_{2}^{D}=c_{3}^{D}=c_{4}^{D}=3\times 10^{-8}$, $c_{5}^{D}=c_{6}^{D}=c_{7}^{D}=c_{8}^{D}=4\times 10^{-8}$, $c_{9}^{D}=c_{10}^{D}=c_{11}^{D}=5\times 10^{-8}$	$c_{1}^{D}=\cdots =c_{7}^{D}=3\times 10^{-8}$, $c_{8}^{D}=\cdots =c_{15}^{D}=4\times 10^{-8}$, $c_{16}^{D}=\cdots =c_{20}^{D}=5\times 10^{-8}$
Computation cost $c^{C}$	$c_{1}^{C}=c_{2}^{C}=c_{3}^{C}=c_{4}^{C}=2\times 10^{-8}$, $c_{5}^{C}=c_{6}^{C}=c_{7}^{C}=c_{8}^{C}=6\times 10^{-8}$, $c_{9}^{C}=c_{10}^{C}=c_{11}^{C}=8\times 10^{-8}$	$c_{1}^{C}=\cdots =c_{7}^{C}=2\times 10^{-8}$, $c_{8}^{C}=\cdots =c_{15}^{C}=6\times 10^{-8}$, $c_{16}^{C}=\cdots =c_{20}^{C}=8\times 10^{-8}$
Delay threshold $Q_{\mathrm{th}}$	1000	1000

## Data Availability

Data sharing is not applicable to this article as no new data were created or analyzed in this study.

## Appendix A

**Proof of Lemma 1.** First, we consider the case of *t* for a fixed $\left(l_{\Delta },s,d\right)$, i.e., appending $A\in F^{T\times S}$ with arbitrary rows of zeros. We prove by contradiction. For a fixed $\left(l_{\Delta },s,d\right)$, suppose the optimal value of *t*, i.e., $t^{*}$, takes values in $\left\{T+1,T+2,\cdots \right\}$. This means that in the padded matrix, denoted as $A_{1}$, there is at least an entire row of blocks, whose values are all $0_{t_{0}\times s_{0}}$. We may remove this entire row of zero blocks and obtain another matrix, denoted as $A_{2}$. We will show that the cost at the user is smaller if we use $A_{2}$ in place of $A_{1}$. This means that it is suboptimal to have the padded matrix contain an entire row of zero small blocks. In other words, $t^{*}$ can not take values in $\left\{T+1,T+2,\cdots \right\}$. To see that the optimal cost at the user is smaller, if we use $A_{2}$ in place of $A_{1}$, we note that both $A_{2}$ and $A_{1}$ have the same dimension for the blocks, i.e., $t_{0}\times s_{0}$. The difference is that $A_{2}$ has a lower number of blocks, more specifically, *t* for $A_{2}$ is less. Any J feasible for the problem of $A_{1}$ is feasible for the problem of $A_{2}$, as the security constraint (10) is the same for both problems due to the fixed $\left(l_{\Delta },s,d\right)$, and the storage constraint (13) and the delay constraint (16) are the same due to both problems having the same dimension for the blocks, i.e., $t_{0}\times s_{0}$. The decodability constraint (11) is more stringent for the problem of $A_{1}$ as *t* is larger for $A_{1}$. Hence, any J feasible for the problem of $A_{1}$ is feasible for the problem of $A_{2}$. Furthermore, the same J that are feasible for both problems incur the same cost (17) due to both problems having the same dimension for the blocks, i.e., $t_{0}\times s_{0}$. Therefore, since the feasibility set of the problem for $A_{2}$ is larger, the optimal cost at the user is smaller. The same argument can be made for *d* for a fixed $\left(l_{\Delta },s,t\right)$. Hence, the proof is complete. □
